# Remineralizing Effects of Resin-Based Dental Sealants: A Systematic Review of In Vitro Studies

**DOI:** 10.3390/polym14040779

**Published:** 2022-02-17

**Authors:** Maha Ibrahim AlGhannam, Mana’a Saleh AlAbbas, Jumanah Abdulla AlJishi, Muath Abdulrahman AlRuwaili, Jehan AlHumaid, Maria Salem Ibrahim

**Affiliations:** 1 College of Dentistry, Imam Abdulrahman Bin Faisal University, Dammam 31441, Saudi Arabia; 2170001326@iau.edu.sa (M.I.A.); 2160006146@iau.edu.sa (M.S.A.); jumanah.aljishi@gmail.com (J.A.A.); 2160004214@iau.edu.sa (M.A.A.); 2 Department of Preventive Dental Sciences, College of Dentistry, Imam Abdulrahman Bin Faisal University, Dammam 34212, Saudi Arabia; jaalhumaid@iau.edu.sa

**Keywords:** bioactive sealant, remineralization, systematic review, caries prevention

## Abstract

The incorporation of remineralizing additives into sealants has been considered as a feasible way to prevent caries by potential remineralization through ions release. Thus, this systematic review aimed to identify the remineralizing additives in resin-based sealants (RBS) and assess their performance. Search strategies were built to search four databases (PubMed, MEDLINE, Web of Science and Scopus). The last search was conducted in June 2020. The screening, data extraction and quality assessment were completed by two independent reviewers. From the 8052 screened studies, 275 full-text articles were assessed for eligibility. A total of 39 laboratory studies matched the inclusion criteria. The methodologies used to assess the remineralizing effect included microhardness tests, micro-computed tomography, polarized-light microscopy, ions analysis and pH measurements. Calcium phosphate (CaP), fluoride (F), boron nitride nanotubes (BNN), calcium silicate (CS) and hydroxyapatite (HAP) were incorporated into resin-based sealants in order to improve their remineralizing abilities. Out of the 39 studies, 32 studies focused on F as a remineralizing agent. Most of the studies confirmed the effectiveness of F and CaP on enamel remineralization. On the other hand, BNN and CS showed a small or insignificant effect on remineralization. However, most of the included studies focused on the short-term effects of these additives, as the peak of the ions release and concentration of these additives was seen during the first 24 h. Due to the lack of a standardized in vitro study protocol, a meta-analysis was not conducted. In conclusion, studies have confirmed the effectiveness of the incorporation of remineralizing agents into RBSs. However, the careful interpretation of these results is recommended due to the variations in the studies’ settings and assessments.

## 1. Introduction

For many countries, oral diseases are considered to be a health burden because they affect people throughout their life, causing pain, discomfort and defacement. According to the Global Burden of Disease Study 2017, oral diseases affect around 3.5 billion people globally, with caries of permanent teeth being the most frequent condition [1].

Dental caries are one of the most prevalent oral diseases. They are caused by interaction between bacterial acids and fermentable carbohydrates. The bacterial acids produced from the bacterial biofilm diffuse into the enamel and dentin, causing demineralization. Caries are considered to be a dynamic process that includes cycles of demineralization and remineralization [2,3]. Remineralization is a natural reparative mechanism for non-cavitated lesions. It depends on calcium (Ca) and phosphate (P) ions, with the help of fluoride (F), to create a new surface on existing crystal remnants in the subsurface lesions that remain after demineralization. Thus, F increases Ca and P precipitation, as well as the development of Fluorhydroxyapatite in tooth tissues [4,5].

A white-spot lesion is the earliest form of dental caries. The continuity of the demineralization process will lead to cavitation. Once the cavitation takes place, preventive measures may not be effective [3]. If a good oral environment can be achieved before cavitation, the caries’ progression can be arrested or reversed [6]. Therefore, caries can be prevented when the remineralization process overcomes the demineralization by either reducing pathogenic factors or increasing protective factors [5]. The use of F can reduce the prevalence of dental caries and their progression rate. Thus, preventive and conservative management strategies such as the application of topical F, pit and fissure sealants, and the use of fluoridated toothpaste and mouth-rinses can help in caries prevention [7].

Pits and fissures of occlusal surfaces are more prone to caries, as they act as reservoirs for *Streptococcus mutans* [8]. Dental sealants on deciduous and permanent teeth act as a physical barrier between the pits and fissures and the oral environment. Thus, the pit and fissure sealants can effectively prevent caries and reduce the need for further restorations by inhibiting microorganisms and plaque accumulation [9]. Methyl cyanoacrylate was the first pit and fissure sealant to be introduced in the 1960s by Cueto. However, this sealant was susceptible to bacterial disintegration with time [10]. Afterward, Bowen developed a viscous resin known as BIS-GMA that effectively bonds with etched enamel and overcomes the bacterial disintegration that Cueto suffered [11].

Different materials are used in pit and fissure sealants, such as resin-based sealants (RBS) and glass ionomer (GI) sealants. RBS are categorized into four generations based on their method of polymerization. Nuva-Seal is an example of the first generation, which is polymerized by ultraviolet light. However, it is not used anymore. The second generation of the RBS are chemically cured by adding tertiary amine to their composition [6]. The third generation has a short setting time, as it is polymerized by light [12]. The last generation is the fluoride-releasing RBS. According to the RBS’ viscosity, RBS can be categorized into filled and unfilled sealants. Moreover, it can be categorized into opaque and transparent sealants [13].

The differences in the properties between the materials make the decision making difficult for the practitioner. Therefore, the choice of the appropriate pit and fissure sealants should be based on the patient’s age and behavior, and the timing of the tooth’s eruption [13]. Although RBSs are effective in caries prevention, they are moisture sensitive [14]. Therefore, when a tooth can’t be isolated or is partially erupted, a GI sealant is an alternative choice due to its moisture-tolerance property [15]. Several studies found that the RBS compete with the GI sealants in terms of long-term retention specifically when the application is performed in adequate isolation. However, the resin materials do not have the antibacterial properties and fluoride release that the GI sealants have [16,17]. Studies showed that the incorporation of remineralizing additives such as fluoride and calcium phosphate into RBSs may improve their therapeutic effect and caries prevention [18,19,20,21]. Therefore, the ideal pit and fissure sealants require good mechanical properties with antibacterial and remineralizing effects.

In the field of Dental Biomaterials, in vitro studies are helpful because they allow researchers to develop new materials and evaluate certain clinically relevant properties that may be difficult to evaluate otherwise. Consequently, this type of study may help in the evaluation of the materials’ properties before exposing patients to them and their possible side effects [22]. Thus, this systematic review aimed to summarize the findings of in vitro studies that assessed the remineralizing additives containing RBSs, in order to identify the remineralizing additives in RBSs and assess their remineralizing performance.

## 2. Materials and Methods

### 2.1. Research Question

The Reporting Items for Systematic Reviews and Meta-Analyses (PRISMA) guidelines for systematic reviews and meta-analysis were followed in this review [23]. A pre-determined, unpublished review protocol was used. The review question was “What are the remineralizing effects of RBSs that incorporate remineralizing additives in their compositions?”

### 2.2. Search Strategy

Comprehensive search strategies for four electronic databases were developed and performed by three authors (M.I.A., M.S.A. and M.S.I.). On 1 June 2020, PubMed, Web of Science, SCOPUS and OVID were queried for published records regardless of their language and date. The four searches resulted in a total of 4920, 2626, 2039, and 2518 potentially relevant references. The search strategies were explained in detail in a previously published review from group [24]. The databases were searched for keywords, text words and subject terms related to the remineralization effects of RBS.

### 2.3. Inclusion and Exclusion Criteria

The articles included in this review were in vitro studies that assessed the remineralization activities of RBS either by microhardness tests, micro-computed tomography or polarized-light microscopy (lesion depth). Moreover, studies that assessed ion-releasing ability and acid neutralization by pH changes were included. Meanwhile, studies that were not laboratory studies, intervention other than sealants, studies that did not have an RBS, studies that only assessed resin-modified glass ionomers, and studies that didn’t assess remineralizing activities were excluded.

### 2.4. Study Screening and Selection

The screening process was performed by three independent reviewers who were not blind to the identity of the authors or journal of the studies. The procedure included a title and abstract screening, then a full-text screening. A senior reviewer resolved disagreements among the reviewers (M.S.I.).

### 2.5. Data Extraction

The data were extracted by two independent reviewers using a customized data collection form. Qualitative and quantitative data were extracted from the included studies. The following data were extracted: details of the studied materials, sample size per group, sample type, curing type, remineralizing agent, and control and intervention groups. The outcomes including microhardness, lesion depth, acid neutralization and ion-releasing ability were also extracted.

### 2.6. Quality Assessment

The studies were assessed for their methodological quality by two independent reviewers (M.I.A. and M.S.I.) using a well-accepted quality assessment tool adapted from several published studies [25,26]. The sampling bias was appraised by assessing whether a study reported the sample size, and whether the samples underwent preparation and randomization. The sample preparation was reported when the study mentioned how the samples were cleaned and prepared. Moreover, the assessment bias was appraised by assessing whether a study had a control group, blind examiners, and more than one assessment method. The reporting bias was described when the study didn’t mention definitive values after the outcome measurements. However, in a study that utilized only qualitative measurement methods, the definitive value was not applicable. The studies were considered to have a low risk of bias when they contained one to three parameters. Studies containing four to five parameters were considered to have a medium risk of bias. Meanwhile, there was a high risk of bias when the studies had six to seven parameters.

### 2.7. Data Synthesis

Qualitative summaries of the included studies’ characteristics, assessment methods and findings were planned to be reported. A meta-analysis was planned to be conducted if no methodological heterogenicity or interventional heterogeneity were found.

## 3. Results

### 3.1. Study Selection

**Figure 1:**
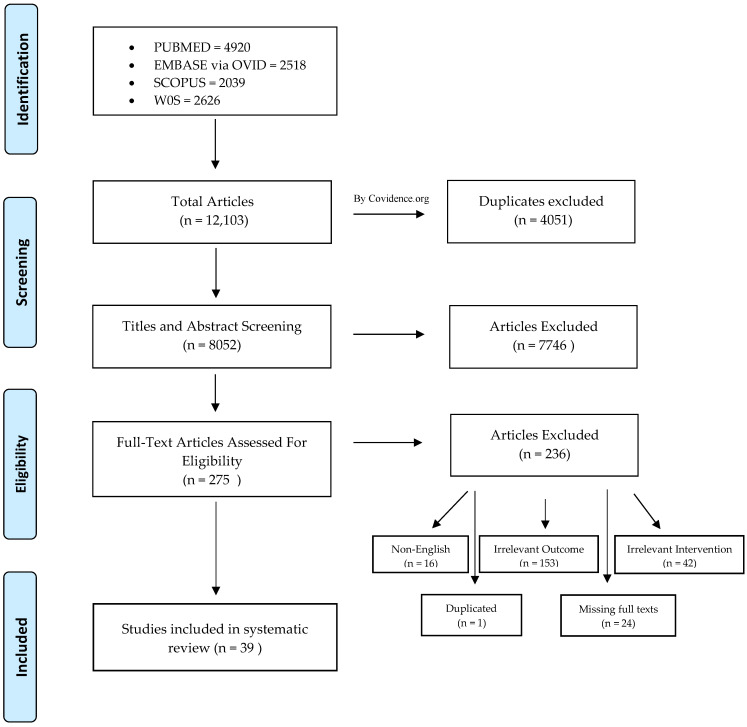
Flow diagram of the study screening and selection.

From the four databases (PubMed, OVID, SCOPUS and WOS), 12,103 studies were identified as being potentially relevant. Duplicated studies were removed. Thus, 8052 studies remained for the title and abstract screening. After the determination of the inclusion criteria and abstract screening, 7746 articles were excluded. Two hundred and fifty-seven studies were assessed for eligibility and full-text screening. A total of 39 in vitro studies that focused on the remineralizing activity of resin-based materials were included in this systematic review. This process is presented in Figure 1.

### 3.2. Risk of Bias Appraisal

**Figure 2:**
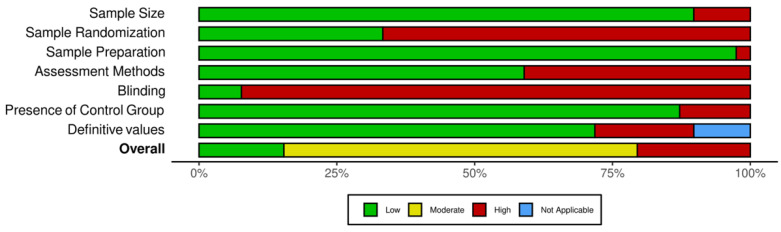
Overall risk of bias for each parameter.

Most of the included studies showed a moderate risk of bias overall (Table 1). Only six studies out of the thirty-nine included studies were judged to have a low risk of bias [27,28,29,30,31,32] (Table 2). Randomization and blinding were not reported in most of the included studies, leading to a positive risk of bias (Figure 2). Almost all of the included studies reported the sample size per group and the sample preparation details.

### 3.3. Study Characteristics

Table 2 summarizes the characteristics of the 39 included studies. In general, the sample type was varied between the use of human non-carious teeth or samples made from the tested materials. Generally, most of the studied materials were light-cured, except for a few studies which used chemically cured materials [18,28,39,42,56,58,60]. The remineralizing agents in the tested materials included F, amorphous calcium phosphate (ACP), bioactive glass, strontium (Sr), hydroxyapatite (HAP), calcium silicate (CS), boron nitride nanotubes (BNNT), and calcium phosphate (CaP). The studies assessed the remineralizing abilities of the sealants using different methods, such as scanning electron microscopy (SEM) with energy dispersive X-ray (EDX) analysis, polarized light microscopy analysis, and the measurement of the hardness change, surface roughness, acid neutralization, ion release, and lesion depth. Some studies assessed the general material properties, such as the flexural strength, curing depth, degree of conversion, surface free energy and color.

### 3.4. Remineralization Findings

Seven studies assessed the remineralizing abilities of the tested materials by measuring the hardness change [19,28,29,32,33,52,57]. There was a variation in the pH cycling method. Three studies used a 5-day cycle [28,32,33], one study used a 20-day cycle [52], and one study used a 4-day cycle [19] for pH cycling. All of the included studies that assessed the hardness change showed a significant difference between the remineralizing sealants and the non-remineralizing sealants, except for two studies [19,32]. However, when the hardness was measured only for the material without measuring the baseline and the change in the hardness, it was considered a physical property, and was not included in this review. Furthermore, the reminreliaizng abilities was assessed using SEM-EDX analysis in two studies [33,44], and seven studies used SEM imaging [20,27,35,36,48,51,54]. There was a variation in the results between the studies. Some of the studies showed that there were significant differences, and some showed no significant differences in the remineralizing abilities of the tested materials. Moreover, only six studies used PLM to assess the remineralizing abilities [30,31,33,37,45,57]. nACP containing a sealant, Pro-seal, Guardian Seal^TM^, Fuji VII^TM^ and GC Fuji Triage sealants showed a thinner enamel lesion. Moreover, only two studies assessed the remineralization using surface roughness. The BNNT-containing sealants and Clinpro sealants showed significantly lower roughness than the control groups [35,58]. Lastly, acid neutralization was used in two studies to measure the remineralization potential. The incorporation of CS, hCS and BAG into the RBS showed significantly higher acid-neutralization abilities [34,47]. A summary of the remineralization findings is given in Table 3.

### 3.5. Ions Release Findings

Out of the 39 included studies, almost 23 studies assessed F ion release. Mostly, the studies showed that the F stopped releasing or declined dramatically after a few days (7–9 days), which indicates a short-term release. Furthermore, it was observed that the GI-based sealants released more F than the RBS. Besides F, Ca and P ion release was assessed in a few studies, and it was observed that the release of these ions lasted longer than the F (21–70 days) [27,34,40]. Furthermore, a few studies assessed Sr, sodium (Na), aluminum (Al), silicon (Si) and boron (B) ion release [49,51]. It was noticed that these ions’ release was significantly high in the bioactive RBS (BeautiSealant) [20,38,43,49,51]. Nevertheless, one study reported that BeautiSealant released the lowest amount of fluoride [18], and another study stated that there was no significant difference between BeautiSealant and Teethmate F-1 sealants [20]. A summary of the ion release outcome findings is presented in Table 4.

## 4. Discussion

Remineralizing agents have been incorporated into the composition of RBSs in order to improve their therapeutic bioactivity. This review included 39 laboratory in vitro studies that assessed the remineralization abilities of RBSs. The aim of this review was to map and summarize these studies, in order to help future in vitro studies to establish uniform laboratory protocols, and to translate the knowledge from the bench to the clinic.

Eight out of the thirty-nine included studies showed a high risk of bias, twenty-five showed a moderate risk of bias, and only six studies showed a low risk of bias. In general, it was observed that there were deficiencies in the areas of randomization and blinding. Randomization is well known in elimination bias through the use of the probability theory, and in maintaining a certain level of sample blinding [62]. It is suggested that future studies control these types of bias by using randomization and blinding whenever they are possible.

Different remineralizing agents were incorporated into the RBSs in order to improve their therapeutic bioactivity. Out of 39 studies, 32 studies focused on F as a remineralizing agent. Furthermore, bioactive glass, ACP, Sr, HAP, CS, BNNT and CaP were incorporated into RBSs. The effectiveness of F and CaP on enamel remineralization was confirmed in most of the included studies. BNNT and CS, on the other hand, had a small or insignificant effect on remineralization [34,35]. This notwithstanding, more laboratory studies are needed in order to confirm their effectiveness. Furthermore, most of the included studies focused on the short-term effects of these additives. Hence, studies with a longer experimental period may improve the understanding of the long-term effects of these additives.

Two of the included studies used bovine teeth [28,35], and fourteen studies used human teeth to assess the ion release and remineralizing abilities of the studied sealants. The majority used resin discs. The main concern with these findings is that in vitro results may be overestimated or underestimated in terms of their ion release and remineralizing abilities when compared to clinical performance in the dynamic oral environment.

Beyond the fact that most studies included control groups, seven studies did not include any control group (Table S1). Although they frequently produce predictable results, they are an important component of all experiments. Generally, there are two types of control groups: negative and positive controls. The negative control group is expected to demonstrate what occurs when the intervention is not applied. On the other hand, the positive control group is the one that is not subjected to the experimental treatment but is instead exposed to another treatment that is known to have a similar effect to the experimental treatment. When the control groups are used correctly, they not only validate the experiment but also offer the foundation for the analysis of the effect of the applied treatments [63]. Hence, they must be treated as any other experimental group in terms of preparation, randomization, blinding and other factors. It is recommended for future studies aiming to evaluate the remineralizing additives in RBSs to use both types of control groups. The positive control group will help as a benchmark for the effectiveness of the experimental treatment. In this vein, studies with this type of control group will aid us in the comparison of the effectiveness of the new RBSs with the conventional ones. Furthermore, the negative control group will help in the determination of the efficacy of the new RBSs in comparison to a lack of treatment.

Most of the included studies did not mention the sample size calculation. Researchers often use previous studies to determine the sample size, with little critical thinking regarding the sample calculation. However, it is critical to optimize the sample size, as it affects the power and impact of the study. For instance, a limited sample size can reduce the statistical power and lead to a type-II error (a false-negative), which occurs when the hypothesis test fails to reject a null hypothesis that is truly false. Furthermore, the larger the sample size, the more time and money is wasted [64]. Therefore, the researchers must be aware of its importance, and a scientific approach must be used to obtain it.

There are multiple qualitative and quantitative assessment methods that can be used to assess the remineralizing activities of resin-based dental sealants, such as tooth samples’ hardness change, SEM-EDX analysis, PLM imaging, lesion depth, and ion release assessment. The included studies showed some variations in this area. Sixteen of the included studies performed only one assessment, while the rest of the studies used more than one assessment to confirm their results. Hence, the use of multiple assessment methods is suggested in order to support the result of each tested materials with a different assessment.

PLM is a qualitative analysis of the mineral contents in the enamel lesions. The change in the backscatter for the enamel can be related to the chemically determined mineral loss [33,65]. As the included studies in this review used PLM to assess the lesions’ depth before and after the application of the sealants, smaller enamel lesions were found in the images when remineralizing sealants were used. This explains why a small amount of demineralization happens on the enamel surface. However, it should be recommended that PLM imaging must be accompanied by a quantitative analysis, such as SEM-EDX [31] or atomic absorption spectroscopy [66], in order to gain a clear description of the mineral volume.

The results showed that the sealants which had remineralizing agents in their compositions had a lower hardness change when compared to the non-remineralizing sealants. However, the protocols to create the lesions may actually affect the material’s performance [33]. The included studies had a maximum of 20 days of pH cycling. How will the performance be affected if the period exceeded that period? Will the materials be able to perform the same, or will we notice a decrease in the surface hardness? As such, we suggest that future studies assess the performance of remineralizing sealants in a longer pH-cycling process in order to ensure the long-term effect of the remineralization.

There was a diversity in the results of the remineralizing abilities when SEM-EDX analysis was used. SEM with EDX analysis is a quantitative analysis used to observe the material elements in a high-resolution image. One of the included studies [33] assessed the mineral content of teeth treated with different types of sealants after pH-cycling. It used PLM, which showed less demineralization around the enamel, and then it supported the results by SEM-EDX, which showed higher calcium and phosphate levels in the enamel.

In this review, an ion release test was performed in more than half of the included studies (26 studies). It was observed that the protocol varied between the studies (Table S2). The variations were observed in the immersion solution, the immersion time, and the pH of the solution. For instance, one study immersed the samples for only 1 day [51], while one study reached up to 180 days [60]. Furthermore, some studies used lactic acid as an immersion solution [34,56]. However, most of the studies used distilled water. These variations may affect the ion release findings. Therefore, standardization in the protocol is recommended in future studies in order to make fair comparisons between the studies.

The prolonged release of remineralizing ions over time from the sealant is required in order to optimize the probability of caries prevention, particularly in individuals at a high risk of caries [67]. Notwithstanding the foregoing, in almost all of the studies, the highest amount of fluoride release was observed on the first day, and then trended to decrease dramatically with time, which indicates a short-term effect. However, Ca and P ions showed longer promising effects regarding ion release [27,34,40]. Due to the fact that fluoride has a short-term release that decreases over time, recharging the dental materials with fluoride has been suggested as a way to maintain a constant amount of fluoride release [68,69]. However, only a few studies [18,32,39,41,46,49,53,61] assessed the fluoride recharging abilities of these sealants. Hence, it is suggested that we perform more studies to confirm the benefits of recharging in these sealants. Furthermore, the incorporation of other remineralizing agents that have longer promising effects, such as those containing Ca and P ions, could be another solution.

Only one of the new, commercially available, bioactive RBSs (BeautiSealant) was studied in the included in studies [18,20,38,43,49,51]. It was observed that this bioactive RBS released multiple ions, such as Na, Sr, Al, Si and B, which contributed to its strong enamel remineralization effect [49,51]. However, it is recommended that we study the other new bioactive dental sealants which have recently been introduced to the dental market in both laboratory and clinical studies.

After the qualitative analysis of the included studies, it was not possible to conduct a quantitative analysis. A meta-analysis was not conducted due to the methodological heterogeneity between the included studies. The careful interpretation of these results is recommended due to the variations of the studies’ settings, experimental protocols and assessment methods.

## 5. Conclusions

In summary, according to the findings of the included in vitro studies, the incorporation of remineralizing agents into RBSs may have promising remineralizing effects which may enhance the therapeutic effect of these sealants. However, this effect seems to diminish over time, and recharging via mouthwashes or toothpastes that contain remineralizing agents may be necessary in order to prolong the effect. For more homogenous studies and a lower risk of bias, a standardized protocol to follow while attempting an in vitro study is recommended.

## Figures and Tables

**Table 1:** Risk of bias appraisal.

Study	Sampling Bias			Assessment Bias			Reporting Bias	Overall
	Sample Size	Sample Randomization	Sample Preparation	Assessment Methods	Blinding	Presence of Control Group	Definitive Values	
**Ibrahim et al., 2020** [33]	+	+	+	+	-	+	-	Moderate
**Yang et al., 2020** [34]	-	-	+	+	-	+	-	High
**Bohns et al., 2019** [35]	+	-	+	+	-	+	NA	Moderate
**Bohns et al., 2019** [36]	+	-	-	+	-	+	NA	High
**Sadrabad et al., 2019** [37]	+	+	+	-	-	+	+	Moderate
**Şişmanoğlu et al., 2019** [38]	+	-	+	-	-	+	+	Moderate
**Khudanov et al., 2018** [39]	+	-	+	-	-	+	+	Moderate
**Ibrahim et al., 2018** [40]	+	-	+	+	-	+	+	Moderate
**Utneja et al., 2018** [27]	+	+	+	+	-	+	+	Low
**Zin EI et al., 2018** [28]	+	+	+	+	-	+	+	Low
**Kosior et al., 2017** [21]	+	-	+	-	-	-	+	High
**Nakamura et al., 2017** [20]	+	-	+	+	-	+	-	Moderate
**Surintanasarn et al., 2017** [41]	+	-	+	-	-	+	+	Moderate
**Dionysopoulps et al., 2016** [18]	+	-	+	-	-	+	+	Moderate
**Munhoz et al., 2016** [42]	+	-	+	+	-	+	-	Moderate
**Salmerón-Valdés et al., 2016** [43]	+	-	+	-	-	-	+	High
**Zawaideh et al., 2016** [19]	+	+	+	-	-	+	+	Moderate
**Hojjati et al., 2014** [44]	+	+	+	+	-	+	NA	Moderate
**Abdel-Haffiez et al., 2013** [45]	+	+	+	-	-	+	+	Moderate
**Fan et al., 2013** [46]	-	-	+	+	-	+	+	Moderate
**Kantovitz et al., 2013** [29]	+	+	+	+	+	+	+	Low
**Yang et al., 2013** [47]	-	-	+	+	-	+	-	High
**Choudhary et al., 2012** [48]	+	+	+	-	-	+	NA	Moderate
**Prabhakar at el., 2012** [30]	+	+	+	-	+	+	+	Low
**Shimazu et al., 2011** [49]	+	-	+	-	-	-	+	High
**Kaga et al., 2011** [50]	+	-	+	+	-	+	-	Moderate
**Wang et al., 2011** [51]	+	-	+	+	-	+	-	High
**AlSaffar et l., 2010** [52]	+	+	+	-	-	+	+	Moderate
**Bayrak et al., 2010** [53]	+	-	+	-	-	+	+	Moderate
**Shen et al., 2010** [54]	+	-	+	-	-	-	+	High
**Kuşgöz et al., 2010** [55]	+	-	+	+	-	+	+	Moderate
**Motohashi et al., 2010** [56]	+	-	+	+	-	+	+	Moderate
**Silva et al., 2010** [57]	+	-	+	+	-	-	+	Moderate
**Cildir et al., 2007** [58]	+	-	+	+	-	+	+	Moderate
**Salar et al., 2007** [31]	+	+	+	+	+	+	+	Low
**Lobo et al., 2005** [32]	+	+	+	+	-	+	+	Low
**Loyola-Rodriquez et al., 1996** [59]	-	-	+	+	-	+	+	Moderate
**Roberts et al., 1984** [60]	+	-	+	-	-	+	+	Moderate
**Swartz et al., 1976** [61]	+	-	+	+	-	+	+	Moderate

+ Yes; - no.

**Table 2:** Characteristics of the included studies.

Study	Sample Type	Sample Size per Group	Curing Type	Remineralizing Agent	Assessed Outcomes
Ibrahim et al., 2020 [33]	Human, non-carious third molars	8	Light cure	nACP	- Hardness change - Scanning electron microscopy with energy-dispersive X-ray spectrometer (SEM-EDX) analysis - Polarized-light microscopy (PLM) imaging
Yang et al., 2020 [34]	Bar-shaped sample (25 mm × 2 mm × 2 mm)	Not mentioned	Light cure	Hydrated calcium silicate	- Acid neutralization - Calcium ion release - Flexural strength - Curing depth - Sorption and solubility
Bohns et al., 2019 [35]	**Surface roughness**: Bovine incisors (5 mm × 5 mm × 2 mm) **Mineral deposition**: Discs-shaped sample (4 mm × 2 mm)	**Surface roughness**: 6 **Mineral deposition**: 3	Light cure	BNNT	- Degree of conversion - Tensile strength - Contact angle - Surface free energy - Surface roughness - color assessment - SRB cytotoxicity assay - Mineral deposition - Cell culture - Scanning electron microscopy (SEM) imaging
Bohns et al., 2019 [36]	**Mineral deposition**: Discs-shaped sample (4 mm × 2 mm)	1	Light cure	- Calcium phosphates - HAP - Tricalcium phosphate - Octacalcium phosphate	- Degree of conversion - SRB cytotoxicity assay - Tensile strength - Mineral deposition - color assessment - SEM imaging
Sadrabad et al., 2019 [37]	Human, non-carious first and second premolars.	8	Light cure	Fluoride	PLM imaging
Şişmanoğlu et al., 2019 [38]	Discs-shaped sample (5 mm × 2 mm)	7	Light cure	Fluoride	Fluoride ions release
Khudanov et al., 2018 [39]	Discs-shaped sample (8 mm × 1.8 mm)	10	- Argecem: chemical cure - Fisskhim: chemical cure - Fissurelight: light cure - Helioseal F: light cure - Helioseal: light cure	Fluoride	- Fluoride ions release - Fluoride ions recharge
Ibrahim et al., 2018 [40]	Bar-shaped samples (2 mm × 2 mm × 25 mm)	1	Light cure	- nACP	- Calcium and Phosphate ions release - Calcium and Phosphate ions recharge - Flexural strength - Flexural modulus - Flowbility assesment
Utneja et al., 2018 [27]	**Remineralization potential**: Human, non-carious maxillary first premolars. **Ion release**: Discs-shaped sample (5 mm × 2 mm)	**Remineralization**: 5 **Ion release**: 9	Light cure	- nACP - HAP - Fluoride	- Micro-shear bond strength - Curing depth - Degree of conversion - SEM imaging - Calcium and Phosphate ions release
Zin EI et al., 2018 [28]	Bovine incisors (7 mm × 7 mm × 2 mm)	10	- Teethmate F-1: light cure - Clinpro: light cure - G bond Plus: light cure - Estelite flow Quick: light cure - Fuji VII: chemical cure	Fluoride	- Fluoride ions release - Hardness change - Optical Coherence Tomography
Kosior et al., 2017 [21]	Cylinders-shaped sample	3	Light cure	Fluoride	Fluoride ions release
Nakamura et al., 2017 [20]	**Mineral loss:** Human, non-carious deciduous molars. **pH changes & Ions release:** Bar-shaped sample (3 mm × 6 mm × 6 mm)	12	Light cure	- S-PRG - Fluoride - Strontium	- Mass and pH changes - Fluoride and Strontium ions release - SEM imaging
Surintanasarn et al., 2017 [41]	Discs-shaped sample (10 mm × 1 mm)	10	Light cure	Fluoride	- Fluoride ions release - Fluoride ions recharge
Dionysopoulps et al., 2016 [18]	Cylinders-shaped samples (7 mm × 2 mm)	8	- Teethmate F-1: light cure - Fissurit F: light cure - BeautiSealant: light cure - FX-II: chemical cure	Fluoride	- Fluoride ions release - Fluoride ions recharge
Munhoz et al., 2016 [42]	Cylinders-shaped sample (4 mm × 6 mm)	4	- ALPHA SEAL– AUTO: chemical cure - ALPHA SEAL–LIGHT: light cure - VITRO SEAL ALPHA: light cure - VITRO FIL: chemical cure	Fluoride	- Fluoride ions release - Tensile strength - color assessment - Flowbility assesment
Salmerón-Valdés et al., 2016 [43]	Discs-shaped sample: (5 mm × 1 mm)	8	Light cure	Fluoride	Fluoride ions release
Zawaideh et al., 2016 [19]	Human, non-carious third molar	25	Light cure	- Fluoride - ACP	Hardness changes
Hojjati et al., 2014 [44]	Human, permanent maxillary/mandibular premolar teeth	7	Light cure	β-tricalcium phosphate (β-TCP)	- Flexural strength - Flexural modulus - Micro-shear bond strength - SEM-EDX analysis
Abdel-Haffiez et al.,2013 [45]	Human, premolars	20	Light cure	Fluoride	PLM imaging
Fan et al., 2013 [46]	Discs-shaped sample (5 mm × 1.2 mm)	1	Light cure	Fluoride	- Fluoride ions release - Fluoride ions recharge - Microtensile bond strength - Microleakage
Kantovitz et al., 2013 [29]	Human, non-carious third molars (4 mm × 4 mm × 2 mm)	12	Light cure	Fluoride	- Hardness change - Marginal adaptation - PLM imaging
Yang et al., 2013 [47]	Bar-shaped sample (25 mm × 2 mm × 2 mm)		Light cure	45S5 Bioactive glass (BAG)	- Acid neutralization. - Flexural strength - Sorption and solubility
Choudhary et al., 2012 [48]	Human, non-carious maxillary first premolar	10	Light cure	- ACP - Fluoride	SEM imaging
Prabhakar at el., 2012 [30]	Human, non-carious third molars	20	Light cure	Fluoride	PLM imaging
Shimazu et al., 2011 [49]	Discs-shaped sample (15 mm × 1 mm)	5	Light cure	Fluoride	- Fluoride ions release - Fluoride ions recharge - Si, Sr, Al, B, and Na ions release
Kaga et al., 2011 [50]	Discs-shaped sample (6 mm × 3 mm)	72	Light cure	- Fluoride - S-PRG	- Fluoride ions release - Si, Sr, Al, Ba, B, P and Ca ions release - Tensile strength
Wang et al., 2011 [51]	Discs-shaped sample (13 mm × 1 mm)	4	Light cure	- Fluoride - S-PRG	- SEM imaging - Fluoride ions release - Si, Sr, Al, B, and Na ions release - pH change
AlSaffar et l., 2010 [52]	Human, non-carious mandibular molars and third molars	10	- Delton: light cure - UltraSeal XT plus: light cure - Clinpro: light cure - Bosworth Aegis: light cure - Fuji Triage: dual cure	- Fluoride - ACP	- Mineral loss - Hardness change
Bayrak et al., 2010 [53]	Discs-shaped sample (10 mm × 1 mm)	10	Light cure	Fluoride	- Fluoride ion release - Fluoride ion recharge
Shen et al., 2010 [54]	Discs-shaped sample (10 mm × 2 mm)	30	Light cure	Fluoride	- Fluoride ion release - Chlorhexidine release - SEM imaging
Kuşgöz et al., 2010 [55]	Discs-shaped sample (5 mm × 2 mm)		- Clinpro: light cure - Grandio Seal: light cure - Fuji Triage: dual cure	Fluoride	- Degree of conversion - Hardness - Microleakage - Fluoride ions release
Motohashi et al., 2010 [56]	Discs-shaped sample (5 mm × 2 mm)	4	- Teethmate-F1: light cure - FujiIII: chemical cure	Fluoride	- Fluoride ions release - Sorption and solubility
Silva et al., 2010 [57]	Bar-shaped sample (4 mm × 4 mm × 2 mm mm)	40	Light cure	- Fluoride - ACP	- Hardness change - Fluoride ion release - PLM imaging
Cildir et al., 2007 [58]	**Surface roughness**: Discs-shaped sample (8 mm × 2 mm)	5	- Clinpro: light cure - Embrace: light cure - Fuji VII: chemical cure - Ketac Molar: chemica cure	Fluoride	- Fluoride ions release - Surface roughness - Compressive strength
Salar et al., 2007 [31]	Human, non-carious third molar	15	- Delton: light cure - ProSeal: light cure - Fuji Triage: dual cure	Fluoride	PLM analysis
Lobo et al., 2005 [32]	Human, non-carious third molar	12	Light cure	Fluoride	- Fluoride ions release - Fluoride ions recharge - Hardness change
Loyola-Rodriquez et al., 1996 [59]	Discs-shaped sample (3 mm × 3 mm)	Not mentioned	Light cure	Fluoride	- Antibacterial activities - Fluoride ions release
Roberts et al., 1984 [60]	**Part 1** Discs-shaped sample **Part 2**: Human, non-carious mandibular molars and maxillary premolars	Part 1: 6 Part 2: 8	Chemical cure	Fluoride	Fluoride ions release
Swartz et al., 1976 [61]	Discs-shaped sample (9.5 mm × 2.2 mm)	8	Light cure	Fluoride	- Fluoride ions release - Fluoride ions recharge - Sorption and solubility - Tensile strength - Hardness - Bond strength - Microleakage

ACP: amorphous calcium phosphate; Amorphous calcium phosphate; HAP: Hydroxyapatite; BNNT: Boron-nitride nanotubes; S-PRG: Surface reaction-type pre-reacted glass ionomer; nACP: Nano-amorphous calcium phosphate.

**Table 3:** Remineralization ability findings.

Assessment Method	Study	pH-Cycling Protocol	Studied Groups (Mean ± SD)			Summary of Results
Hardness Change	Ibrahim et al., 2020 [33]	- DE: Prepared solution, 6 h, pH = 4.7 - RE: Prepared solution, 18 h, pH = 7.0 - For 5 Days	- nACP: 65.3 ± 5.6% - nACP + DMAHDM: 60.9 ± 6.5 % - Negative control: Reaching the 100% (Estimated from the graph)			The nACP containing sealants showed a significantly lower SHL% in comparison to the negative control group (*p* < 0.05).
	Zin EI et al.,2018 [28]	- DE: Prepared solution, pH = 4.8 - For 5 Days	- G bond Plus: NDV - Teethmate F-1: NDV - Clinpro: NDV - Fuji VII: NDV			Teethmate F-1 showed a significantly higher SHL% in comparison to all the other groups (*p* < 0.05).
	Zawaideh et al., 2016 [19]	- DE: Prepared solution, pH = 5 - For 4 Days	- Concise: 117.78 ± 10.22 - Aegis^®^: 93.50 ± 10.22 - Conseal-FTM: 24.28 ± 10.12			There were no statistically significant differences between all the groups (*p* > 0.05).
	Kantovitz et al., 2013 [29]	- DE: Prepared solution, 16 h, pH = 5.5 - RE: Artificial saliva solution, 7 Days, pH = 7.0	**Under sealants**: - SF: 6364 ± 3967 - SH: 5584 ± 3788 - CF: 3763 ± 2549 - CH: 5408 ± 2657 - CFF: 5033 ± 3448 - CFH: 7474 ± 3455 **Sealant margin**: - SF: 6682 ± 4127 - SH: 8579 ± 5181 - CF: 6022 ± 3669 - CH: 10,856 ± 10,825 - CFF: 6385 ± 4286 - CFH: 8556 ± 3463	**100 μm Outer sealant**: - SF: 7084 ± 5412 - SH: 7239 ± 5495 - CF: 6421 ± 3859 - CH: 9662 ± 4331 - CFF: 6533 ± 4246 - CFH: 8631 ± 3404 **200 μm Outer sealant**: - SF: 4901 ± 3822 - SH: 7841 ± 5197 - CF: 5443 ± 3813 - CH: 8322 ± 3831 - CFF: 6782 ± 4655 - CFH: 8467 ± 2511		FluroShield sealant showed a significantly lower SHL% in comparison to the Helioseal sealant (*p* < 0.05).
	AlSaffar et al., 2010 [52]	- DE: Prepared solution, pH = 5.1 - For 20 Days	- Delton Opaque: l975 ± 806% - UltraSeal XT plus: 1802 ± 512% - Clinpro: 1004 ± 421% - Bosworth Aegis: 1275 ± 375% - GC Fuji Triage: 88 ± 124%			Clinpro, Bosworth Aegis and GC Fuji Triage sealants showed significantly lower SHL% in comparison to the Delton Opaque and UltraSeal XT plus (*p* < 0.05).
	Silva et al., 2010 [57]	DE: Prepared solution, 16 h, pH = 5	- Fluroshield: NDV - Aegis: NDV - Experimental sealant containing fluoride (ESF): NDV - Experimental sealant containing ACP and fluoride (ACP-F): NDV			Aegis, Fluroshield and ESF sealants had higher surface microhardness and %SMHR values than ACP-F sealant.
	Lobo et al., 2005 [32]	- DE: Prepared solution, 6 h, pH = 4.3 - RE: Prepared solution, 18 h, pH = 7.0 - For 5 Days	- No Sealant: NDV - Vitremer: NDV - Clinpro: NDV - Concise: NDV			There were no statistically significant differences between all the groups in the hardness of the sealed enamel (*p* > 0.05).
SEM-EDX Analysis	Ibrahim et al., 2020 [33]	- DE: Prepared solution, 6 h, pH = 4.7 - RE: Prepared solution, 18 h, pH = 7.0 - For 5 Days	- PMGDM:NDV-EBPADMA: NDV - HEMA: NDV - Bis-GMA: NDV - BAPO: NDV			The nACP-containing sealant showed higher weight percent of Ca and P in comparison to the negative group (*p* < 0.05).
	Hojjati et al., 2014 [44]	- DE: Prepared solution, pH = 5 - For 4 Days	- 1 wt% b-TCP-NPs: NDV - 2 wt% b-TCP-NPs: NDV - 3 wt% b-TCP-NPs: NDV - 4 wt% b-TCP-NPs: NDV - 5 wt% b-TCP-NPs: NDV - Concise: NDV - Control: NDV			Increasing the concentrations of β-TCP decreased the enamel irregularities/crack lines due to demineralization. Sealants containing 4% and 5% of β-TCP showed a homogenous layer at the enamel-sealant interface.
Polarized light Imaging	Ibrahim et al., 2020 [33]	- DE: Prepared solution, 6 h, pH = 4.7 - RE: Prepared solution, 18 h, pH = 7.0 - For 5 Days	- Negative Control: NDV - Experimental sealant containing nACP: NDV			The nACP-containing sealant showed a thinner enamel lesion in comparison to the control group.
	Sadrabad et al., 2019 [37]	- DE: Artificial saliva solution, 3 h, pH = 4.5 - RE: Prepared solution, 2 h, pH = 7.0 - For 10 Days	**Primary caries:** - Embrac Wetbond: 603.12 ± 51.73 - Master dent: 889.37 ± 56.38 - Negative control: 1438.75 ± 138.12 **Final caries:** - Embrac Wetbond: 30 ± 32.24 - Master dent: 419.37 ± 258.84 - Negative control: 647.18 ± 175.08			There were statistically significant differences between all the groups (*p* ˂ 0.001).
	Abdel-Haffiez et al., 2013 [45]	- DE: Artificial saliva solution, 1 h, pH = 4.4 - For 35 Days	- Pro-Seal: NDV - Fluor Protector: NDV - Negative control: NDV			Pro-seal sealant showed a thinner enamel lesion in comparison to the control groups and fluoride varnish sample.
	Prabhakar et al., 2012 [30]	DE: Acidified gelatin gel, 1008 h	- Helioseal: NDV - Guardian SealTM: NDV - GC Fuji VIITM: NDV			Guardian Seal^TM^ and Fuji VII^TM^ sealants showed a thinner enamel lesions in comparison to Helioseal sealant.
	Silva et al., 2010 [57]	DE: Prepared solution, 16 h, pH = 5	- Fluroshield: NDV - Aegis: NDV - Experimental sealant containing fluoride: NDV - Experimental sealant containing ACP and fluoride: NDV			Fluroshield sealants and the experimental sealant containing fluoride showed a thinner enamel lesions in comparison to Aegis sealant.
	Salar et al., 2007 [31]	- DE: Artificial saliva solution, pH = 4.25 - RE: Solution, pH = 7 - For 42 Days	- ProSeal: 144 ± 21 - GC Fuji Triage: 128 ± 15 - Delton: 232 ± 17			ProSeal and GC Fuji Triage and Fuji VIITM sealants showed a thinner enamel lesion in comparison to Delton sealant.
Surface Roughness	Bohns et al., 2019 [35]	- DE: Artificial saliva solution, pH = 4.5 - For 28 Days	- Sound: 0.86 ± 0.28 - Demineralized: 3.06 ± 1.00 - Control group: 2.36 ± 0.58 - 0.1%BNNT: 2.42 ± 0.60 - 0.2%BNNT: 2.44 ± 0.49			Sound enamel, 0.1% BNNT sealant and 0.2% BNNT sealant showed significantly lower surface roughness in comparison to the demineralized enamel and control group (*p* < 0.05).
	Cildir et al., 2007 [58]	Not mentioned	**Day1**: - Clinpro: 0.050 ± 0.015 - Embrace: 0.071 ± 0.012 - Fuji VII: 0.193 ± 0.014 - Ketac Molar: 0.182 ± 0.024	**Day 28**: - Clinpro: 0.081 ± 0.032 - Embrace: 0.082 ± 0.012 - Fuji VII: 0.224 ± 0.016 - Ketac Molar: 0.196 ± 0.040	**Day70**: - Clinpro: 0.108 ± 0.030 - Embrace: 0.109 ± 0.027 - Fuji VII: 0.404 ± 0.033 - Ketac Molar: 0.341 ± 0.09	Clinpro sealant showed significantly lower surface roughness in comparison to Fuji VII sealant (*p* < 0.0001).
Acid neutralization	Yang et al., 2020 [34]	- DE: Prepared solution, pH 4 - For 28 Days	- hCS 50.0: 11.99 ± 0.19 - hCS 37.5: 11.30 ± 0.05 - hCS 25.0: 10.02 ± 0.14 - hCS 12.5: 8.03 ± 0.19 - CS 50.0: 11.67 ± 0.05			The pH of CS-containing and hCS-containing groups was significantly higher than hCS0 group (*p* < 0.05).
	Yang et al., 2013 [47]	DE: Prepared solution, pH 4 For 3 h	- BAG0:NDV - BAG12.5: 157.8 ± 22.1 min - BAG25: 92.6 ± 15.7 min - BAG37.5: 48.6 ± 11.6 min - BAG50: 22.6 ± 4.4 min			The BAG50 sealant showed significantly higher acid neutralization in comparison to all the groups (*p* < 0.05).
SEM Imaging	Bohns et al., 2019 [35]	- DE: Artificial saliva solution, pH = 4.5 - For 28 Days	- Sound: NDV - Demineralized: NDV - Control group: NDV - 0.1%BNNT: NDV - 0.2%BNNT: NDV			There were no statistically significant differences between all the groups (*p* > 0.05). After 28 days in the media, SEM images showed minerals deposition over the BNNT-containing sealants.
	Bohns et al., 2019 [36]	- DE: Artificial saliva solution - For 28 Days	- Sound: NDV - Demineralized: NDV - S_HAP_: NDV - S_α-TCP_: NDV - S_OCP_: NDV - Control group: NDV			After 7 days of immersion in artificial saliva, minerals deposition was observed on the surface of sealants containing-TCP and HAP. After 28 days in the media, SEM images showed minerals deposition over SHAP sealants samples. Phosphate peak showed high intensity.
	Utneja et al., 2018 [27]	- DE: Prepared solution 3 h - RE: Prepared solution 2 h - For 10 Days	- Unfiled sealant 0% filler: NDV - 30 wt% nHAP filled sealant: NDV - 10% nHAP + 20% n silica filled sealnt: NDV - 10% nHAP + 20% nACP filled sealnt: NDV - Delton FS plus: NDV - Aegis: NDV - Clinpro: NDV			The sealants containing HAP had a homogeneous white remineralized area at the tooth surface sealant interface, which was more noticeable in the 30% nHAP filled sealant. Aegis and Delton FS plus sealants had a white irregular globular zone at the tooth surface sealant interface. Clinpro and the prepared unfilled sealants had no white remineralized zone.
	Nakamura et al., 2017 [20]	- DE: Prepared solution, 2 min, pH = 4.5 - RE: Prepared solution, 3 min, pH = 7 - For 35 Days	- Teethmate F-1: NDV - BeautiSealant: NDV - Fuji III LC: NDV			BeautiSealant and FujiIILC sealants showed lower demineraliztion, and the enamel-surfaces were smoother than the teathmate F-1 sealant.
	Choudhary et al., 2012 [48]	- DE: Artificial saliva solution, 24 h, pH = 4.0 - For 14 Days	- Aegis- Opaque: NDV - Teethmate F1: NDV - Concise- Opaque: NDV			Concies sealant showed lower demineralization than the Ageis and Teathmate F-1 sealants.
	Wang et al., 2011 [51]	DE: Lactic acid solution, 24 h, pH = 4.0	- BeautiSealant: NDV - DELTON FS: NDV - Teethmate F-1: NDV - Fuji lll LC: NDV - Control: NDV			BeautiSealant and DELTON FS sealants showed lower demineralization than the Teathmate F-1, Fuji lll LC sealants and control group.
	Shen et al., 2010 [54]	- DE: Prepared solution, pH = 4, 5, 6 - For 120 days	- 2Ca/ 8CHX: NDV - 5Ca/5CHX: NDV - 8Ca/2CHX: NDV			There was no significant difference between the groups (*p* > 0.05). Chlorohexidine release was higher when pH decreased.

DE: Demineraliztion; RE: Remineralization; NDV: No definitive values were given; nACP: Nano-amorphous calcium phosphate; DMAHDM: Dimethyla-minohexadecyl methacrylate; SHL: Surface hardness loss; SF: Sound + FluroShield; SH: Sound + Helioseal clear chroma; CF: Caries-like lesion + FluroShield; CH: Caries-like lesion + Helioseal clear chroma; CFF: Caries + topical fluoride + FluroShield; CFH: Caries + topical fluoride + Helioseal clear chroma; PMGDM: Pyromellitic glycerol dimethacrylate; EBPADMA: Ethoxylated bisphenol A dimethacrylate ; HEMA: 2-Hydroxyethyl methacrylate; Bis-GMA: Bisphenol A glycidyl dimethacrylate; BAPO: Phenyl-bis (2,4,6- trimethyl benzoyl)-phosphine oxide; BNNT: Boron-nitride nanotubes; hCS: Hydrated calcium silicate; CS: Calcium silicate; S_HAP_: Sealant with Hydroxyapatite; S_α-TCP_: Sealant with α-tricalcium phosphate; S_OCP_: Octacalcium phosphate; nHAP: Nano-hydroxyapatite; CHX: Chlorhexidine.

**Table 4:** Ion release findings.

Study	Studied Groups (Mean ± SD)		Summary of Results
Yang et al., 2020 [34]	- hCS 0: NDV - hCS 12.5: NDV - hCS 25.0: NDV - hCS 37.5: NDV - hCS 50.0: NDV - CS 50.0: NDV		The hCS 37.5, hCS 50.0, and CS 50.0 sealants showed the highest amount of calcium ions release on day 1 then declined dramatically over the immersion time. The hCS 50.0 sealant showed significantly higher initial calcium ions concentration than other groups (*p* < 0.05)
Şişmanoğlu et al., 2019 [38]	**Day 1**: - BeautiSealant: 5.33 ± 0.67 ppm - Clinpro: 2.69 ± 0.43 ppm - HelioSeal F: 2.91 ± 0.64 ppm - Fissurit F: 2.94 ± 0.67 ppm	**Day 28**: - BeautiSealant: 1.12 ± 0.02 ppm - Clinpro sealant: 1.00 ± 0.06 ppm - HelioSeal F: 1.01 ± 0.03 ppm - Fissurit F: 1.21 ± 0.03 ppm	For all materials, the highest amount of fluoride ions release was seen on the first day. BeautiSealant group released the highest amount of fluoride ions on the first two days (*p* < 0.05). There were no significant differences between Clinpro, Fissurit F and HelioSeal F sealants on day 1 (*p* > 0.05).
Khudanov et al., 2018 [39]	- Argecem: 125.24 ± 12.45 μg/cm^2^ - Fisskhim: 1.71 ± 1.18 μg/cm^2^ - Fissulight: 0.67 ± 0.13 μg/cm^2^	- Helioseal F: 7.93 ± 0.81 μg/cm^2^ - Helioseal: 0.78 ± 0.17 μg/cm^2^	The highest amount of fluoride ions was seen on the first day then decreased with time until recharge. The highest amount of released fluoride ions was seen in Argecem sealant and the least in Helioseal sealant
Ibrahim et al., 2018 [40]	**Calcium**: - 30% nACP + 5% DMAHDM: 4.70 ± 0.95 mmol/L - 20% nACP + 5% DMAHDM: 3.64 ± 0.11 mmol/L.	**Phosphate**: - 30% nACP + 5% DMAHDM: 4.25 ± 0.12 mmol/L - 20% nACP + 5% DMAHDM: 3.41 ± 0.10 mmol/L	After 70 days of ions release, 30% nACP + 5% DMAHDM sealant released a higher amount of calcium and phosphate ions than 20% nACP + 5% DMAHDM sealant (*p* < 0.05).
Utneja et al., 2018 [27]	**Calcium**: **Day 21 at pH 4**: - 10% nHAP + 20% nACP: 1.02 ± 0.108 mmol/L. - Aegis: 0.88 ± 0.012 mmol/L. **Day 21 at pH 5.5**: - 10% nHAP + 20% nACP: 0.65 ± 0.077 mmol/L. - Aegis: 0.47 ± 0.028 mmol/L. **Day 21 at pH 7.4**: - 10% nHAP + 20% nACP 0.23 ± 0.009 mmol/L. - Aegis: 0.20 ± 0.004 mmol/L	**Phosphate**: **Day 21 at pH 4**: - 10% nHAP + 20% nACP: 0.55 ± 0.085 mmol/L - Aegis: 0.41 ± 0.035 mmol/L. **Day 21 at pH 5.5**: - 10% nHAP + 20% nACP: 0.27 ± 0.021 mmol/L - Aegis: 0.23 ± 0.020 mmol/L. **Day 21 at pH 7.4**: - 10% nHAP + 20% nACP: 0.13 ± 0.010 mmol/L. - Aegis: 0.10 ± 0.007 mmol/L.	The 10% nHAP + 20% nACP filled sealant showed a higher amount of calcium and phosphate ions release at pH 4 compared to the commercial sealant with ACP (Aegis).
Zin EI et al., 2018 [28]	**Day 14**: - Fuji VII: 69.5 ± 12 μg/cm^2^ - Teethmate F-1: 7.26 ± 2.13 μg/cm^2^ - Clinpro: 3.94 ± 0.9 μg/cm^2^		For all materials, the highest fluoride release was observed after 2 days. FVII sealant released the greatest amount of fluoride followed by the Teethmate F-1and Clinpro sealants. Among all sealants, there were significant differences in fluoride concentrations with different time intervals (*p* < 0.05).
Kosior et al., 2017 [21]	**Day 1**: - Delton FS+:11.4 ± 3.16 μg/mm^2^ - Fissurit FX: 8.08 ± 1.28 μg/mm^2^ - Conseal F: 5.31 ± 1.46 μg/mm^2^ - Admira Seal: 0.65 ± 0.3 μg/mm^2^	**Week 14**: - Delton FS+:61.91 ± 12.07 μg/mm^2^ - Fissurit FX: 28.08 ± 3.10 μg/mm^2^ - Conseal F: 19.83 ± 2.80 μg/mm^2^ - Admira Seal: 7.36 ± 0.30 μg/mm^2^	For all materials, the highest fluoride release was observed after 1 h.The highest level of ions release was seen on Deltion FS+, followed by Fissurit FX, Conseal F and Admira Seal sealants.
Nakamura et al., 2017 [20]	- Teethmate F-1: NDV - BeautiSealant: NDV - Fuji III LC: NDV		The amount of fluoride and strontium ions released from Fuji III LC was significantly higher than BeautiSealant and Teethmate F-1 sealants. On week 1, BeautiSealant and Teethmate F-1 sealants were not significantly different (*p* > 0.05). There were significant differences in Strontium ions release between all groups (*p* < 0.05).
Surintanasarn et al., 2017 [41]	**Day 3**: - Mesoporous silica: ND - Calcium carbonate: ND - fluoro-alumino silicate: 0.1024 ± 0.0077 ppm - Control: ND	**Day 27**: - Mesoporous silica: ND - Calcium carbonate: ND - fluoro-alumino silicate: ND - Control: ND	On day 3 and 6, initial fluoride release was seen only in RBS with 5% of fluoro-alumino silicate glass. For all groups, fluoride levels on day 9 were at baseline.
Dionysopoulos et al., 2016 [18]	- Teethmate-F1: 89.45 ± 12 μg/cm^2^ - Fissurit F: 68.62 ± 8.72 μg/cm^2^ - BeautiSealant: 33.32 ± 4.91 μg/cm^2^ - FX-II: 408.56 ± 45.66 μg/cm^2^		The highest fluoride ions release was observed in the fluoridated materials after day 1. FX-II sealant released significantly more fluoride than the other materials (*p* < 0.05) while the BeautiSealant group were the lowest. There was a significant difference in fluoride ions release between the materials (*p* < 0.05).
Munhoz et al., 2016 [42]	- Vitro Fil: NDV - Alpha Seal Auto: NDV - Alpha Seal Light: NDV - Vitro Seal Alpha: NDV		Vitro Fil released the highest amount of fluoride. There were no significant differences between Alpha Seal Auto, Alpha Seal Light and Vitro Seal Alpha groups (*p* < 0.05).
Salmerón-Valdés et al., 2016 [43]	**Day 1**: - BeautiSealant: 5.1 ± 1.1 ppm - Clinpro: 2.7 ± 0.6 ppm - Helioseal: F: 3.0 ± 1.0 ppm - UltraSeal XT: 4.8 ± 1.1 ppm	**Day 60**: - BeautiSealant: 1.02 ± 0.0 ppm - Clinpro: 1.0 ± 0.0 ppm - Helioseal F: 1.0 ± 0.0 ppm - UltraSeal XT (US) plus: 1.0 ± 0.0 ppm	For all materials, the highest amount of fluoride ions was observed on the first day and then declined until day 60. There were significant differences in fluoride ions release between the materials (*p* < 0.005).BeautiSealant group showed the highest fluoride ions release while Clinpro sealant was the lowest.
Fan et al., 2013 [46]	- Clinpro: NDV - FluoroShield: NDV - SeLECT Defense: NDV	- Sealant containing 35% Fluoride-releasing Filler: NDV - Sealant containing 20% Fluoride-releasing Filler + 15% Bioactive Glass: NDV	Sealant containing 35% Fluoride-releasing Filler and sealant containing 20% Fluoride-releasing Filler + 15% Bioactive Glass showed the highest fluoride release in comparison to Clinpro sealant (*p* < 0.005). No fluoride ions release was observed in SeLECT Defense sealant.
Shimazu et al., 2011 [49]	**Fluoride:** **Day 1**: - BeautiSealant: 12.60 ± 1.19 ppm - Delton FS+: 45.80 ± 5.46 ppm - Teethmate F-1: 4.66 ± 0.82 ppm	**Day 25**: - BeautiSealant: 15.84 ± 3.25 ppm - Delton FS+: 4.24 ± 0.35 ppm - Teethmate F-1: 0.96 ± 0.24 ppm - **Na, Sr, Al, Si and B ions release**: NDV	There were significant differences between the sealants on day 1 (*p* < 0.001). All materials showed a decrease in fluoride ions release on day 2. Increase in fluoride ions release presented in BeautiSealant group on days 16,19, 22 and 25. The BeautiSealant showed significant increase in the release of Sodiom (Na), Strontium (Sr), Aluminum (Al), Silicon (Si), and Boron (B) ions.
Kaga et al., 2011 [50]	- S-PRG filler containing pit and fissure sealant: NDV - Delton FS+: NDV - Fujji lll LC: NDV - Teethmate F-1 2.0: NDV		For all sealants, the highest amount of fluoride ions was observed in the first week then dropped dramatically in the second week. Fujji III LC sealant showed the highest amount of fluoride ions release at all time periods (*p* < 0.05), while Teethmate F-12.0 sealant released the smallest amount from third week to the end of the test period.
Wang et al., 2011 [51]	- BeautiSealant: NDV - Delton FS+: NDV - Fujji lll LC: NDV	- Teethmate F-1 2.0: NDV - Silica oxide filler: NDV	BeautiSealant groups showed significant release of Si, Sr, Al, B, Na and F, while Fuji lll LC group released less (*p* < 0.05). Fuji lll LC sealant showed greater fluoride release than of BeautiSealant and Delton FS+ sealants.Teethmate F-1 2.0 sealant released the smallest amount of fluoride.
Bayrak et al., 2010 [53]	**Day 1**: - Fuji VII: 213.65 ±43.34 μg/mm^2^ - Fuji II LC: 99.50 ± 7.43 μg/mm^2^ - Fissurit F: 50.84 ± 8.40 μg/mm^2^ - Ionosit: 10.64 ± 2.56 μg/mm^2^ - Aelite Flo: 0.82 ± 0.25 μg/mm^2^	**Day 21**: - Fuji VII: 17.07 ± 9.66 μg/mm^2^ - Fuji II LC: 21.41 ± 0.755 μg/mm^2^ - Fissurit F: 1.38 ± 0.11 μg/mm^2^ - Ionosit: 0.30 ± 0.05 μg/mm^2^ - Aelite Flo: 0.13 ± 0.01 μg/mm^2^	For all materials, the highest amount of fluoride ions release was seen on the first day then decreased dramatically. GI sealants released higher amount of fluoride ions than the other materials (*p* < 0.05). There were significant differences in fluoride ions release between the materials (*p* < 0.05).
Shen et al., 2010 [54]	**Fluoride**: - 2Ca/8CHX: 120 ± 11 μg/cm^2^ - 5Ca/5CHX: 272 ± 44 μg/cm^2^ - 8Ca/2CHX: 252 ± 33 μg/cm^2^ - 2Ca/8CHX: 79 ± 9 μg/cm^2^	- 5Ca/5CHX:243 ± 53 μg/cm^2^ - 8Ca/2CHX: 241 ± 73 μg/cm^2^ - 2Ca/8CHX: 73 ± 13 μg/cm^2^ - 5Ca/5CHX: 208 ± 25 μg/cm^2^ - 8Ca/2CHX: 213 ± 28 μg/cm^2^	When the pH of the media decreased, the CHX and fluoride ions release increased. Fluoride salt decreased the chlorhexidine release where the chlorhexidine significantly increased the fluoride ions release.
Kuşgöz et al., 2010 [55]	**Day 1**: - Grandio Seal: 4.56 ± 0.18 μg/cm^2^ - Clinpro: 6.47 ± 0.07 μg/cm^2^ - Fuji Triage: 957.2 ± 4.45 μg/cm^2^	**Day 30**: - Grandio Seal: 47.83 ± 1.7 μg/cm^2^ - Clinpro: 58.18 ± 4.08 μg/cm^2^ - Fuji Triage: 2698 ± 22 μg/cm^2^	Fuji Triage group showed the highest fluoride ions release at all the periods when compared to Clinpro and Grandio Seal groups *(p* < 0.05).Clinpro released fluoride more than Grandio seal with no significant difference between the two groups (*p* > 0.05).
Silva et al., 2010 [57]	- Control: NDV - Fluroshield: NDV - Aegis: NDV - Experimental sealant containing F (ESF) NDV - Experimental sealant containing (ACP-F): NDV		The highest amount of fluoride ions release was observed in experimental and Fluroshield sealants with no significant differences between them (*p* > 0.05). The highest amount of calcium ions release was observed in Fluroshield, The highest amount of phosphate ions release was observed in the control group which differed significantly from the other groups (*p* < 0.05). The lowest amount was observed in ACP-F sealant which was statistically like Aegis (*p* > 0.05).
Motohashi et al., 2010 [56]	- FujiIII: NDV - Teethmate-F1: NDV		FujiIII sealant significantly released more fluoride ions than Teethmate-F1 sealant.
Cildir et al., 2007 [58]	- FujiVII: NDV - Ketac Molar: NDV - Clinpro: NDV - Embrace: NDV		The highest amount of fluoride ions was released during the first day then dropped dramatically on the second day. There were significant differences between the RBSs and GICs (*p* < 0.0001). Fuji VII group exhibited the highest amount of fluoride ions release (*p* < 0.0001) followed by Ketac Molar, while Clinpro sealant showed the lowest amount of fluoride ions release.
Lobo et al., 2005 [32]	- Vitremer:1.91 (0.53) μg F/mL - Clinpro: 0.12 (0.17) μg F/mL - Concise: 0.07 (0.17) μg F/mL		The amount of fluoride released during pH-cycling did not change significantly between Clinpro and Concise groups (*p* < 0.01). Vitremer group released the highest amount of fluoride ions (*p* < 0.01).
Loyola-Rodriquez et al., 1996 [59]	**Day 1**: - Teethmate-F: 231 ± 12 ppm/mg - Fluoroshield: 209 ± 13 ppm/mg - Helioseal: 0 ± 0 ppm/mg	**Day 7**: - Teethmate-F: 124 ± 05 ppm/mg - Fluoroshield: 25 ± 2 ppm/mg - Helioseal: 0 ± 0 ppm/mg	For all sealants, the highest amount of fluoride ions was released in the first two days then gradually decreased to around 50% release after 7 days. Teethmate-F sealant released the highest amount of fluoride ions.
Roberts et al., 1984 [60]	**Day 1**: - 0.00% sodium fluoride: 0.60 μg - 0.1% sodium fluoride: 4.75 μg - 0.25% sodium fluoride: 13.05 μg - 1.0% sodium fluoride: 61.35 μg - 2.5% sodium fluoride: 95.55 μg	**Day 91–180**: - 0.00% sodium fluoride: 0.005 μg - 0.1% sodium fluoride: 0.010 μg - 0.25% sodium fluoride: 0.015 μg - 1.0% sodium fluoride: 0.035 μg - 2.5% sodium fluoride: 0.040 μg	The highest amount of fluoride was released during the first day then dropped dramatically. Resin containing 2.5% sodium fluoride showed the highest fluoride ions release per day at all time periods except 91-180 days.
Swartz et al., 1976 [61]	- Nuva Seal: NDV - Epoxylite: NDV - BIS-GMA resins: NDV - isobutyl cyanoacrylate resin: NDV		The amount of fluoride ions released by the isobutyl cyanoacrylate resin was significantly more than the other three sealants.

hCS: Hydrated calcium silicate; CS: Calcium silicate; nACP: Nano-amorphous calcium phosphate; DMAHDM: Dimethylaminohexadecyl methacrylate; nHAP: Nano-hydroxyapatite; S-PRG: Surface reaction-type pre-reacted glass ionomer; CHX: Chlorhexidine; NDV: No definitive values were given; ND: Not detectable (<0.01 ppm).

## Data Availability

No new data were created or analyzed in this study. Data sharing is not applicable to this article.

## Supplementary Materials

The following are available online at https://www.mdpi.com/article/10.3390/polym14040779/s1, Table S1: The details of the control and intervention groups of all included studies, Table S2: The details of the protocol of all included studies.
