# Supplementary material for: Remineralizing Effects of Resin-Based Dental Sealants: A Systematic Review of In Vitro Studies
Source: Polymers (Basel). 2022 Feb 17;14(4):779. doi: 10.3390/polym14040779 (PMC8877949; doi:10.3390/polym14040779)
Supplement: Supplementary file 1 [file polymers-14-00779-s001.zip › polymers-1556654-SM.pdf]

S1. The details of the control and intervention groups of all included studies.		
**Study**	**Control Group**	**Intervention Group**
Ibrahim et al., 2020 [33]	- Negative Control (no sealant application)<br>- Control Sealant (base formulation)<br>50 wt% PEHB (44.5% PMGDM + 39.5 EBPADMA + 10% HEMA + 5% Bis-GMA + 1% BAPO) + 50 wt% Glass	- 50 wt% PEHB + 20 wt% nACP + 30 wt% Glass<br>- 45 wt% PEHB + 20 wt% nACP + 30 wt% Glass + 5 wt% DMAHDM
Yang et al., 2020 [34]	hCS 0: 50 wt% RM + 0 hCS Filler + 0 wt% CS Filler + 50 wt% silanized glass<br><br>RM: 49.5 wt % Bis-GMA + 49.5 wt % TEGDMA, + 0.3 wt% CQ, + 0.6 wt % 2(DMAEMA)	- hCS 12.5: (50 wt% RM + 12.5 wt% hCS + 0 wt% CS + 37.5 wt% salinized glass)<br>- hCS 25.0: (50 wt% RM + 25 wt% hCS + 0 wt% CS + 25 wt% salinized glass)<br>- hCS 37.5: (50 wt% RM + 37.5 wt% hCS + 0 wt% CS + 12.5 wt% salinized glass)<br>- hCS 50.0: (50 wt% RM + 50 wt% hCS + 0 wt% CS + 0 wt% Salinized glass)<br>- CS 50.0: (50 wt% RM + 0 wt% hCS + 50 wt% CS + 0 wt% salinized glass)
Bohns et al., 2019 [35]	S_{CG}: 90 wt% TEGDMA + 10 wt% Bis-GMA + 1 mol% CQ and 1 mol% EDAB	- SBNNT0.1%: 90 wt% TEGDMA + 10 wt% Bis-GMA + 1 mol% CQ and 1 mol% EDAB + 0.1% Hexagonal BNNTs<br>- SBNNT0.2%: 90 wt% TEGDMA + 10 wt% Bis-GMA + 1 mol% CQ and 1 mol% EDAB + 0.2% Hexagonal BNNT
Bohns et al., 2019 [36]	S_{CG}: 90 wt% (TEGDMA) + 10 wt% (BisGMA) + 1 mol% (CQ) + 1 mol% (EDAB)	- SHAp: 90 wt% (TEGDMA) + 10 wt% (BisGMA)+ 1 mol% (CQ) + 1 mol% (EDAB) + 10 wt% Ca10(PO4)6(OH)2<br>- Sα-TCP: 90 wt% (TEGDMA) + 10 wt% (BisGMA)+ 1 mol% (CQ) + 1 mol% (EDAB) + 10 wt% α-Ca3(PO4)2<br>- SOCP: 90 wt% (TEGDMA) + 10 wt% (BisGMA)+ 1 mol% (CQ) + 1 mol% (EDAB) + 10 wt% Ca8(HPO4)2(PO4)4·5H2O
Sadrabad et al., 2019 [37]	- Master-Dent<br>- Negative Control (no sealant application)	Embrac Wetbond
Şişmanoğlu et al., 2019 [38]		- Clinpro<br>- Helioseal F<br>- Fissurit F<br>- BeautiSealant
Khudanov et al., 2018 [39]	- Argecem<br>- Helioseal	- Fisskhim<br>- Fissurelight<br>- Helioseal F
Ibrahim et al., 2018 [40]	Virtuoso	- FluroShield<br>- 5% DMAHDM + 0% nACP:<br>- (45% PEHB + 5% DMAHDM + 50% Glass + 0% NACP)<br>- 5% DMAHDM + 10% nACP:

		- (45% PEHB + 5% DMAHDM + 40% Glass + 10% NACP)<br>- 5% DMAHDM + 20% nACP: (45% PEHB + 5% DMAHDM + 30% Glass + 20% nACP)<br>- 5% DMAHDM + 30% nACP: (45% PEHB + 5% DMAHDM + 20% Glass + 30% nACP)
Utneja et al., 2018 [27]	- Clinpro (unfilled)<br>- 35.5 wt% Bis-GMA + 35.5 wt% TEGDMA + 28%wt HEMA + 0.8 wt% 4E + 0.2 wt% CQ + 0 wt% filler (unfilled)	- Delton FS plus<br>- Aegis<br>- 35.5 wt% Bis-GMA + 35.5 wt% TEGDMA + 28%wt HEMA + 0.8 wt% 4E + 0.2 wt% CQ + 30 wt% nHAP filler<br>- 35.5 wt% Bis-GMA + 35.5 wt% TEGDMA + 28%wt HEMA + 0.8 wt% 4E + 0.2 wt% CQ + 10 wt% nHAP+20 wt% silica filler<br>- 35.5 wt% Bis-GMA + 35.5 wt% TEGDMA + 28%wt HEMA + 0.8 wt% 4E + 0.2 wt% CQ + 10 wt% nHAP + 20 wt% nACP filler
Zin EI et al., 2018 [28]	- Fuji VII<br>- G bond Plus<br>- Estelite flow Quick	- Teethmate F-1<br>- Clinpro
Kosior et al., 2017 [21]		- Conseal F<br>- Fissurit FX<br>- Delton FS+<br>- Admira Seal
Nakamura et al., 2017 [20]	- Negative Control (no sealant application)<br>- Fuji III LC	- Teethmate F-1<br>- BeautiSealant
Surintanasarn et al., 2017 [41]	Concise sealant without filler	- Concise sealant + 5 wt% mesoporous silica<br>- Concise sealant + 5 wt% calcium carbonate<br>- Concis sealant + 5 wt% Fluoro-alumino silicate
Dionysopoulps et al., 2016 [18]	FX-II	- Teethmate F-1<br>- Fissurit F<br>- BeautiSealant
Munhoz et al., 2016 [42]	- VITRO FIL<br>- VITRO SEAL ALPHA	- ALPHA SEAL – AUTO<br>- ALPHA SEAL – LIGHT
Salmerón-Valdés et al., 2016 [43]		- BeautiSealant<br>- Clinpro<br>- Helioseal F<br>- UltraSeal XT
Zawaideh et al., 2016 [19]	Concise™	- Aegis<br>- Conseal-F™

Hojjati et al., 2014 [44]	- Concise<br>- Control: 70 wt% BIS-GMA + 30 wt% TEGDMA + 0.5 wt% DMAEMA+ 0.5 wt%CQ + 7 wt% Silanized Silica	- 1 wt% β-TCP-NPs<br>- 2 wt% β-TCP-NPs<br>- 3 wt% β-TCP-NPs<br>- 4 wt% β-TCP-NPs<br>- 5 wt% β-TCP-NPs
Abdel-Haffiez et al.,2013 [45]	- Negative Control (no sealant application)<br>- Fluor Protector	Pro-Seal
Fan et al., 2013 [46]	SeLECT Defense	- Sealant containing 35% Fluoride-releasing Filler: 16.13 wt% Synthesized antibacterial fluoride releasing monomer + 6.45 wt% BisGMA + 12.90 wt% UEDMA + 12.90 wt% HDDMA + 12.90 wt% HEMA + 3.22 wt% synthesized adhesive monomer + 35 wt% silanized fluoride-releasing filler + 0.5 wt% photo- initiators<br>- Sealant containing 20% Fluoride-releasing Filler + 15% Bioactive Glass: 16.13 wt% Synthesized antibacterial fluoride releasing monomer + 6.45 wt% BisGMA + 12.90 wt% UEDMA + 12.90 wt% HDDMA + 12.90 wt% HEMA + 3.22 wt% synthesized adhesive monomer + 20 wt% silanized fluoride-releasing filler + 15 wt% NovaMin bioactive glass + 0.5 wt% photo- initiators<br>- FluroShield<br>- Clinpro
Kantovitz et al., 2013 [29]	- Sound + Helioseal clear chroma (SH)<br>- Caries-like lesion + Helioseal clear chroma (CH)<br>- Caries + topical fluoride + Helioseal clear chroma (CFH)	- Sound + FluroShield (SF)<br>- Caries-like lesion + FluroShield (CF)<br>- Caries + topical fluoride + FluroShield (CFF)
Yang et al., 2013 [47]	- BAG0: 50 wt% RM + 0 wt% 45S5 BAG filler + 50 wt% silanized dental glass filler<br>RM= Bis-GMA/TEGDMA 50/50 (wt%) containing 1% of DMAEMA/CQ 2:1 (wt%)	- BAG12.5: 50 wt% RM + 12.5 wt% 45S5 BAG filler + 37.5 wt% silanized dental glass filler<br>- BAG25: 50 wt% RM + 25 wt% 45S5 BAG filler + 25 wt% silanized dental glass filler<br>- BAG37.5: 50 wt% RM + 37.5 wt% 45S5 BAG filler + 12.5 wt% silanized dental glass filler<br>- BAG50: 50 wt% RM + 50 wt% 45S5 BAG filler + 0 wt% silanized dental glass filler
Choudhary et al., 2012 [48]	Concise	- Aegis.<br>- Teethmate F1
Prabhakar at el., 2012 [30]	- Helioseal<br>- Fuji VII™	- Guardian Seal™
Shimazu et al., 2011 [49]		- BeautiSealant<br>- Deltion FS+<br>- Teethmate F-12.0
Kaga et al., 2011 [50]	Fuji III LC	- S-PRG filler containing FS: UDMA + TEGDMA+ S-PRG filler<br>- Delton FS+

		- Teethmate F-12.0
Wang et al., 2011 [51]	- Control: UDMA + TEGDMA + Silica oxide filler<br>- Fuji III LC	- BeautiSealant<br>- DELTON FS<br>- Teethmate F - 1 2.0
AlSaffar et l., 2010 [52]	- Fuji Triage<br>- Delton Opaque	- UltraSeal XT plus<br>- Clinpro<br>- Aegis
Bayrak et al., 2010 [53]	- Fuji VII<br>- Fuji II LC	- Fissurit F<br>- Ionosit<br>- Aelite Flo
Shen et al., 2010 [54]		- 2Ca/ 8CHX (70 wt% UDMA+30 wt% TEGDMA + CaF2/CHXDA were prepared with weight ratios of 2/8)<br>- 5Ca/5CHX (70 wt% UDMA+30 wt% TEGDMA + CaF2/CHXDA were prepared with weight ratios of 5/5)<br>- 8Ca/2CHX (70 wt% UDMA+30 wt% TEGDMA + CaF2/CHXDA were prepared with weight ratios of 8/28)
Kuşgöz et al., 2010 [55]	Fuji Triage	- Clinpro<br>- Grandio Seal
Motohashi et al., 2010 [56]	- FujiIII<br>- Negative Control (no sealant application)	Teethmate-F1
Silva et al., 2010 [57]		- Fluroshield<br>- Aegis<br>- Experimental sealant containing fluoride<br>- Experimental sealant containing ACP and fluoride
Cildir et al., 2007 [58]	- Fuji VII<br>- Ketac Molar	- Clinpro<br>- Embrace
Salar et al., 2007 [31]	- Delton<br>- Fuji Triage	ProSeal
Lobo et al., 2005 [32]	- Negative Control (no sealant application)<br>- Concise<br>- Vitremer	Clinpro
Loyola-Rodriquez et al., 1996 [59]	Helioseal	- FluoroShield<br>- Teethmate-FIM

Roberts et al., 1984 [60]	Delton + 0.0% Sodium fluoride	- Delton + 0.1% Sodium fluoride<br>- Delton + 0.25% Sodium fluoride<br>- Delton + 1.0% Sodium fluoride<br>- Delton + 2.5% Sodium fluoride
Swartz et al., 1976 [61]		- Nuva Seal<br>- Eposylite<br>- Exp.BIS-GMA<br>- Exp.cyanoacrylate.

ACP: Amorphous calcium phosphate. RM: Resin Matrix. DMAHDM: Dimethylaminohexadecyl methacrylate. PMGDM: Pyromellitic glycerol dimethacrylate. EBPADMA: Ethoxylated bisphenol A dimethacrylate .HEMA: 2-Hydroxyethyl methacrylate. Bis-GMA: Bisphenol A glycidyl dimethacrylate. BAPO: Phenyl-bis (2,4,6- trimethyl benzoyl)-phosphine oxide. hCS: Hydrated calcium silicate. CS: calcium silicate cement. HAP: hydroxyapatite. TEGMA: Triethylene glycol monomethacrylate. UDMA: Urethane dimethacrylate. TEGDMA: Triethylene glycol dimethacrylate. CQ: Camphorquinone. BNNT: Boron-nitride nanotubes. S-PRG: Surface reaction-type pre-reacted glass ionomer. CaPs: Calcium phosphate. 4E: Ethyl 4-N, N-dimethylaminobenzoate. nACP: Nano amorphous calcium phosphate.

S2. The details of the protocol of all included studies.

**Study**	Summary of Protocol
Yang et al., 2020 [34]	- Lactic acid solution<br>- pH=4<br>- Distilled water.<br>- pH=6.5<br>- 28 days
Şişmanoğlu et al., 2019 [38]	- polyethylene vials with 5 ml of deionized water<br>- For 28 days<br>- at 37°C<br>- +
Khudanov et al., 2018 [39]	- Distilled water<br>- 21 days
Ibrahim et al., 2018 [40]	- Sodium chloride<br>- pH=4<br>- 70 days
Utneja et al., 2018 [27]	- Sodium chloride<br>- pH=4, 5.5 and 7.4<br>- 21 days

Zin EI et al., 2018 [28]	- Deionized water<br>- 15 days<br>- +
Kosior et al., 2017 [21]	- Saline<br>- 14 weeks<br>- +
Nakamura et al., 2017 [20]	- Deionized water<br>- 5 weeks<br>- +
Surintanasarn et al., 2017 [41]	- Deionized water<br>- 27 days<br>- +
Dionysopoulos et al., 2016 [18]	- Deionized water<br>- 28 days
Munhoz et al., 2016 [42]	- Deionized water<br>- 15 days
Salmerón-Valdés et al., 2016 [43]	- Deionized water<br>- 60 days<br>- +
Fan et al., 2013 [46]	- Deionized water<br>- 14 days<br>- +
Shimazu et al., 2011 [49]	- Distilled water<br>- 15 days<br>- +
Kaga et al., 2011 [50]	- Distilled water<br>- 84 days<br>- +
Wang et al., 2011 [51]	- Latic acid.<br>- pH=4<br>- 1 day
Bayrak et al., 2010 [53]	- Ultrapure water<br>- 21 days<br>- +

Shen et al., 2010 [54]	- Sodium acetate-acetic acid buffer<br>- PH 4.0, 5.0, and 6.0<br>- 120 days
Kuşgöz et al., 2010 [55]	- Ultrapure water<br>- 21 days<br>- +
Silva et al., 2010 [57]	Not mentioned.
Motohashi et al., 2010 [56]	- lactic acid<br>- pH=5.7<br>- 14 days
Cildir et al., 2007 [58]	- Distilled deionized water<br>- 28 days<br>- +
Lobo et al., 2005 [32]	- Demineralizing solution 6h<br>- pH=4.3<br>- Remineralizing solution 18h<br>- pH=7<br>- 5 days<br>- +
Loyola-Rodriquez et al., 1996 [59]	- Double distilled deionized water<br>- 7 days<br>- +
Roberts et al., 1984 [60]	- Distilled water<br>- 180 days<br>- +
Swartz et al., 1976 [61]	- Distilled water<br>- 4 days

+ : This study measured the ions release at multiple timepoints, but not all timepoints are reported here.
